# Conductance Channels in a Single-Entity Enzyme

**DOI:** 10.1021/acs.jpclett.4c01796

**Published:** 2024-10-21

**Authors:** Rafael Neri Prystaj Colombo, Steffane Q. Nascimento, Frank Nelson Crespilho

**Affiliations:** 1 São Carlos Institute of Chemistry, University of São Paulo (USP), São Carlos, SP 13566-590, Brazil

## Abstract

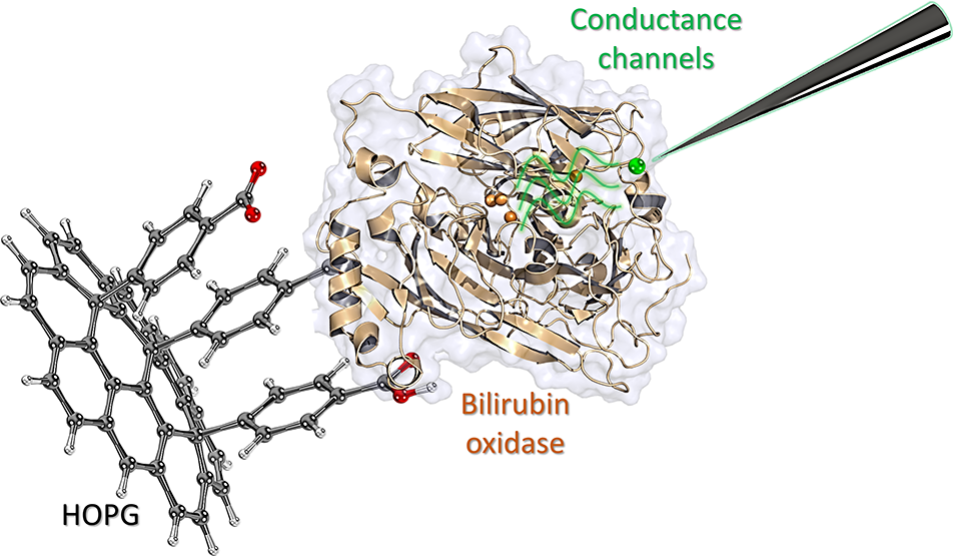

For a long time, the prevailing view in the scientific community was that proteins, being complex macromolecules composed of amino acid chains linked by peptide bonds, adopt folded structure with insulating or semiconducting properties, with high bandgaps. However, recent discoveries of unexpectedly high conductance levels, reaching values in the range of dozens of nanosiemens (nS) in proteins, have challenged this conventional understanding. In this study, we used scanning tunneling microscopy (STM) to explore the single-entity conductance properties of enzymatic channels, focusing on bilirubin oxidase (BOD) as a model metalloprotein. By immobilizing BOD on a conductive carbon surface, we discern its preferred orientation, facilitating the formation of electronic and ionic channels. These channels show efficient electron transport (ETp), with apparent conductance up to the 15 nS range. Notably, these conductance pathways are localized, minimizing electron transport barriers due to solvents and ions, underscoring BOD’s redox versatility. Furthermore, electron transfer (ET) within the BOD occurs via preferential pathways. The alignment of the conductance channels with hydrophilicity maps, molecular vacancies, and regions accessible to electrolytes explains the observed conductance values. Additionally, BOD exhibits redox activity, with its active center playing a critical role in the ETp process. These findings significantly advance our understanding of the intricate mechanisms that govern ETp processes in proteins, offering new insights into the conductance of metalloproteins.

Proteins, composed primarily of semiconjugated amino acid chains linked by peptide bonds and adopting intricate folded structures, initially appear to possess insulating properties. However, this class of biomacromolecules defies convention by exhibiting remarkably high conductance—even reaching nanosiemens (nS) levels—over distances exceeding 10 nm under nonequilibrium conditions.^1−5^ The mechanisms governing electron transport (ETp) in proteins have long puzzled researchers, yet consistent observations of large conductance persist across various experimental and theoretical/simulation approaches.^6−9^

In particular, scanning tunneling microscopy (STM) has played a crucial role shedding light on the importance of protein orientation, conformational ensembles, and the contacts between the substrate–protein and protein–tip.^7,10^ Notably, individual protein molecules exhibit substantially high conductance when measured in aqueous environments, spanning several nanometers.^7^

To explain the conductance within structures lacking conjugated π-bonds, several effects have been postulated.^11^ Aromatic amino acids, such as tryptophan and tyrosine, reduce the reorganization energy to nonequilibrium oxidation states to ca. 0.16 eV. The energy barriers—close to 40 meV—align well with the kinetic thermal energy, contributing to a long-range electron hopping mechanism.^12^ Discussions persist regarding coherent versus decoherent processes, involving sequential tunneling hops within proteins.^13−15^ Possibly, contributions of coherent transport via surface peptide contact leading to the exponential decay factor combine with inelastic transport within the interior of proteins.

In this context, advancements in STM instrumentation have facilitated the coupling of the electronic density interrogation capability with electromagnetic excitation. This development allows for the visualization of quantum coherence phenomena at various scales.^16,17^ Research has demonstrated that correlated protein environments can enable efficient energy transfer by maintaining electronic coherence.^18^ Another important consideration is the role of protein contacts, which can best promote electron transfer through inner contact in proteins—specifically, from an electrode to the hydrophobic interior portion of the structure—rather than through external, more hydrophilic sites.^7^ Notably, when two contacts are established within a protein, the conductance remains constant regardless of the contact distance. In contrast, a nonspecific contact leads to a decay length of less than 10 nm, a value greater than the expected for a hopping mechanism.^3^ Additionally, it has been shown that the vibronic features of metallic ions in metalloproteins, such as with Cu(II) ions, are strongly related to the long-range electron transport, in a mechanism in which tunneling charges traverse the protein.^2^

It can also be speculated that conductance in proteins manifests through ionic and solvent-accessible channels, due to the combination of hydrogen bonds and ionic and water flow, especially to/from active centers containing metallic ions. Assessing the conductance in regions of the channels necessitates isolating a single protein for measurements at a single-entity level. We speculate that these channels serve as passages or pathways through which electrical or ionic current flows with increased coupling compared to the external region. The postulated existence of such channels, coupled with the absence of experimental evidence, motivated us to design an experiment aimed at investigating this phenomenon.

First, we covalently bound the enzyme to a conductive, flat highly oriented pyrolytic graphite (HOPG) substrate, ensuring preferential and stable positioning during measurements (Figure 1A). This allowed us to establish a direct electrical connection with isolated enzymes, avoiding multilayer or aggregates formation and enabling the high-precision analysis of conductance. Next, we employed a setup with sample stabilization including a suspended STM system mounted on a tripod (heavy base stretching bungee cords, *f*_vib_ < 1 Hz) and antivibration pads (see Figure S1 and SI Section 1.5 for additional setup details). This design effectively minimized external disturbances and vibrations, creating a controlled environment for accurate measurements.

**Figure 1:**
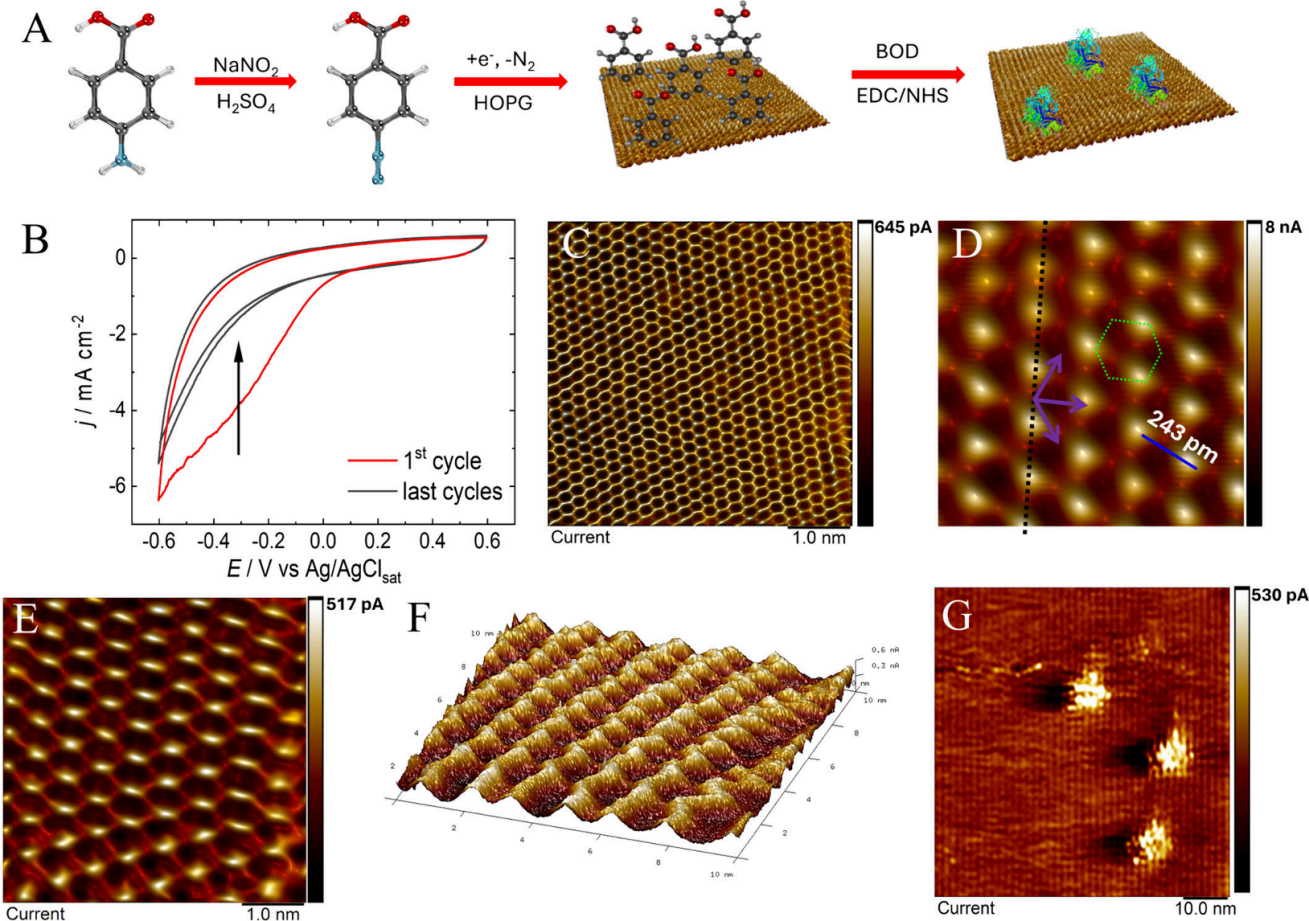
Step-by-step functionalization and characterization of isolated BOD enzymes covalently immobilized onto the HOPG surface. (A) Representative schematic for the benzoic acid chemical functionalization of HOPG through the diazonium salt coupling reaction, followed by the attachment of BOD enzymes through EDC/NHS coupling. (B) Cyclic voltammograms of the HOPG modification with benzoic acid, through diazonium salt chemistry; first cycle in red. (C) STM image of the clean HOPG surface, with lattice vectors of ca. 2.46 A; scale bar = 1 nm, *V*_bias_ = 45 mV, and *I*_s_ = 11 nA. (D) FFT-filtered and zoomed image, with indicated vectors composing the typical HOPG hexagonal patterns and the measured atom-to-atom distance; invisible atoms present due to AB stacking, as in half the hexagon vertices. (E) FFT filtered STM image of HOPG-BA, with lattice vectors of ca. 5.7 A; *V*_bias_ = 75 mV and *I*_s_ = 0.7 nA. (F) Three-dimensional micrograph of HOPG-BA after FFT filtering, showing the ordered pattern with longer lattice vectors, *V*_bias_ = 75 mV. (G) STM micrograph of a region containing three isolated proteins after BOD covalent immobilization through EDC/NHS coupling; *V*_bias_ = 100 mV and *I*_s_ = 0.8 nA.

Lastly, we conducted our experiments in low volumes by using a designed microcell. This setup created an electrolytic environment specifically tailored for studying the enzyme’s conductance properties. By reducing the volume, we minimized unwanted interactions and improved the signal-to-noise (S/N) ratio, thereby enhancing the reliability and accuracy of the measurements.

Our focus is on investigating single-entity enzymes, with bilirubin oxidase (BOD) chosen as the target enzyme due to its role in energy production and biological processes; this protein has also been studied for practical applications when immobilized to surfaces of gold, carbon, and nanostructures including MWCNT.^19−21^ BOD acts as a catalyst for bilirubin oxidation, facilitating electron transfer to molecular oxygen and generating water through the oxygen reduction reaction (ORR).^22^ The active center of BOD consists of localized copper ions, which serve as pivotal relays for electron transfer to electrodes and actively participate in molecular cleavage mechanisms. BOD exhibits unique versatility in ORR and water oxidation reactions, making it a model in bioelectrocatalysis.^23^ We will present evidence and analysis supporting the remarkable observation of high conductance in proteins under nonequilibrium conditions.

To perform single-entity STM experiments, BOD was immobilized on a HOPG surface using a coupling reaction involving EDC/NHS, after the electrochemical formation of an active benzoic acid (BA) layer through diazonium salt chemistry (see FTIR of each step in Figure S2), employing a protocol described elsewhere.^24^Figure 1A illustrates the schematic of the diazonium salt formation and its linkage to HOPG, resulting in a BA layer. Electrochemical modification was performed potentiodynamically with the typical voltammogram shown in Figure 1B. The BA surface coverage (Γ) was estimated via eq 1, by the integrated charge (*Q*), the area (*A*), the number of transferred electrons (*n*), and the Faraday constant (*F*), resulting in ca. 2 × 10^–10^ mol cm^–2^, a similar coverage compared to other works modifying graphitic materials.^24−26^
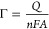
1

This surface was reacted with EDC/NHS, resulting in a chemically active molecular network that facilitates the attachment of the enzymes. The system was explored at the atomic scale using the STM equipment with HOPG serving as the sample electrode and a freshly cut Pt tip as the probe (Figure 1B); the acoustic hood and Faraday cage are not represented. Figure 1C displays a high-resolution STM image of the clean HOPG surface, revealing a well-ordered and regular arrangement of carbon atoms, indicating the high quality and crystalline nature of the surface. The STM images offer a clear view of the electronic properties of the HOPG, facilitating a detailed analysis of its electronic structure and properties. Important parameters, such as the high-resolution carbon lattice structure, enable precise measurements of interatomic distances between neighboring carbon atoms, yielding a calculated lattice constant of approximately 2.46 Å (Figure 1D; additional images in Figure S3). The lattice constant represents a carbon–carbon distance of 142 pm, matching the expected value. STM images clearly depict the hexagonal lattice structure of HOPG (represented in green), revealing the geometry of the lattice, where adjacent C–C bonds in the hexagonal lattice exhibit angles close to 120°. HOPG, with its organized structure, exhibits an A/B stacking sequence and remarkable symmetry; the presence of invisible atoms is a known and well-documented feature.^27^

There are no oxidation side reactions of the sample surface, attributable to the moderate applied tip–sample bias (typically <120 mV). Additionally, no evidence of surface contamination was observed, as all samples were freshly prepared and promptly transferred for analysis.

Figure 1E shows the typical STM image of the surface functionalized with BA after the diazonium salt electrochemical step, revealing a highly ordered and regular arrangement of the functionalized molecules on the surface. This indicates precise control and homogeneity in the functionalization process (functionalization voltammogram and additional images are in Figure S4). The average lattice constant is more than twice that of HOPG, and a horizontal drift is always observed, the cause of which remains unknown. The three-dimensional micrograph shown in Figure 1F, after FFT filtering, emphasizes the regularity of the HOPG–BA surface, indicating the good quality of the functionalization process. The bright regions of high density of states are too far apart to suggest BA molecules parallel to the surface but indeed reinforce perpendicular structures as shown in the Figure 1A schematic; the BA–BA distance onto the carbon sheet is still unknown.

Figure 1G illustrates an area of the HOPG–BA–BOD with three isolated single-BOD enzymes attached to the HOPG surface through prior functionalization; the same surface without chemically attached enzymes are shown in the control in Figure S5. The attempts to find BOD proteins only by adsorption onto HOPG did not lead to attached proteins (Figure S6). These images highlight the controlled attachment of the enzymes through functionalization, with each protein exhibiting a well-defined electronic signature. It is important to note that these results were obtained from replicates, and the data presented are representative of the samples studied. Naturally, surface heterogeneities in the density and disposition of the proteins should be considered, and suitable regions for single-entity analysis are sought during experiments.

The chemical linkage of the BOD to the HOPG through EDC/NHS exhibits a preferential binding at locations rich in lysine/arginine residues, which are more sensitive to the coupling reaction.^28,29^ This is highlighted by the green regions at the bottom, as shown in Figure 2A and Figure S7. Therefore, this observation allows us to estimate the putative preferential orientation of BOD on the carbon surfaces, as schematized by the overlay in Figure 2B. AFM was performed (Figure S8) to check for features and to possibly acquire heigh information, yet the image quality was not good enough due to the large tip sizes compared to the STM ones.

**Figure 2:**
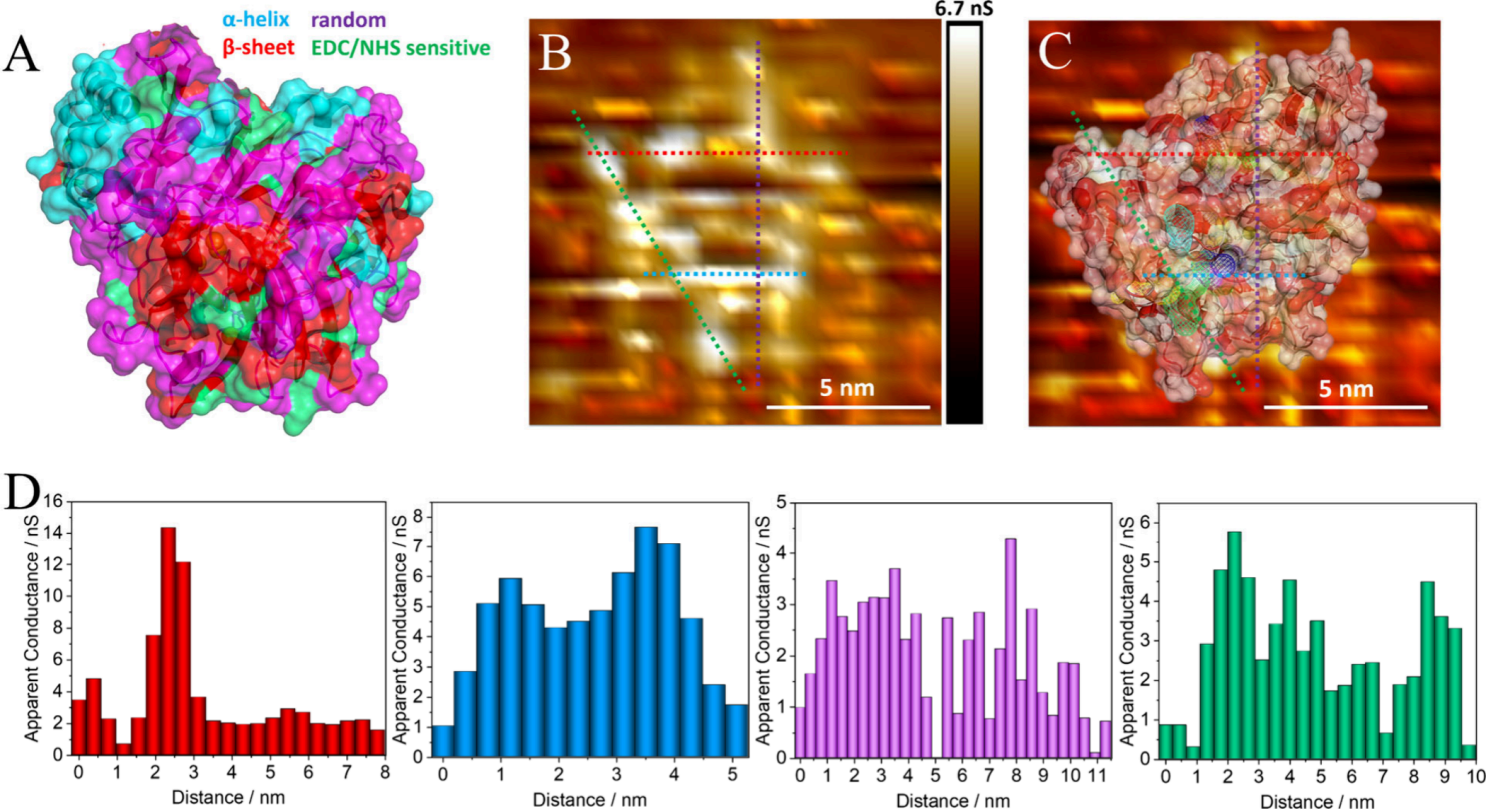
Three-dimensional view of the BOD’s secondary structure, current STM micrographs, and measured local conductance. (A) Orientation of the BOD (PDB code: 2XLL) enzymes, with most sensitive regions to EDC/NHS coupling in green (lysine richer area positioned on the bottom), α-helix regions in cyan, β-sheet regions in red, and random structures in magenta. (B) STM micrograph of an isolated BOD enzyme, with four line measurements; *V*_bias_ = 100 mV and *I*_s_ = 8.5 nA. (C) STM micrograph with enzyme transparent overlay, white regions with higher external hydrophilicity, red regions with higher external hydrophobicity, and access tunnels from surface to the active center highlighted by meshed regions. (D) Apparent conductance measured over selected cross-section profiles represented in (B), as indicated by the respective color.

By combining conductance measurements and molecular orientation observations, we obtain a comprehensive picture of the enzyme’s electronic properties. Conductance channels within BOD, as represented by the meshed cavities of access to the trinuclear copper cluster in Figure 2B, are detected by an algorithm based on Voronoi diagrams and shown in higher details in Figure S7;^30^ these conductance mappings reveal a localized apparent conductance of up to approximately 15 nS, as shown in Figure 2C, with local conductance measurements of the cross sections in Figure 2D (color respective to the sections of Figure 2C). Additional images can be seen in Figure S9.

The micrograph and local conductance measurements suggest that these channels facilitate the passage of a few billion electrons per second. This observation of a high local conductance in a protein aligns with fixed-gap tunneling junction measurements explored for an integrin, with charge transfer in the order of picocoulombs over many milliseconds.^31^ In the isolated BOD enzymes, the conductance channels are distributed in specific spots throughout the protein, particularly coincident to cavities for O_2_ and H_2_O transport to the trinuclear cluster (TNC), and also in an access channel for the Cu_T1_, which is known for the electron-transfer role when electrochemically connected to electrodes;^32,33^ these are an inherent part of the mechanisms for oxygen reduction and water oxidation.^22,23,34^ Particularly, the channels observed promote the transfer of electrons between the T1 and T2/T3 sites, as evidenced by the higher conductance localized above the T1 and the trinuclear cluster (Figure 2B,D). The high apparent conductivity associated with the TNC seems to provide a complementary pathway to the hopping through aromatic residues proposed model from the literature, that is, strongly suggesting that the TNC copper ions participate in the long-range ETp; nonetheless, the contribution of tunneling and hopping in this process cannot be determined solely by the provided data. The correlation of lower tunneling barriers or higher currents in the presence of a metal ion has been noticed in the past, especially in the case of azurin;^35^ however, the exact understanding of how charge transfer occurs is under discussion,^36,37^ and the realization of the role of accessibility channels to the active pockets in these metalloproteins has been unknown.

The importance of hydrophilicity on the external surface is also highlighted by the higher local conductance observed in the more hydrophilic regions of the BOD (white colored); this observation is in conformity to the recent findings of contact-dependence, where electron-transfer through a covalent contact leads to high conductance when (noncovalently) probed at a hydrophilic external surface due to a single barrier.^7^ By comparing STM images with crystallographic data (PBD 2XLL), we can uncover the putative BOD orientation on the HOPG–BA surface. This analysis allowed us to identify polypeptide groups with distinct hydrophilicities within the molecule. The measured distance ratios between specific features align with those obtained from the crystallographic data, indicating a well-preserved structure.

Protein conductance is a complex property that depends on various factors, including the protein’s three-dimensional structure, conformation of amino acid residues, electronic structure of the material, and interaction with the surrounding environment.^4,38^ It is influenced by quantum tunneling, coherence of distinct transport pathways, electronic coupling between amino acid residues, especially the aromatic ones, and effects of solvent and ion interactions.^16,39,40^ Therefore, the conductance of a protein should consider the contribution of different and well-localized regions of the structure and the underlying processes and mechanisms involved in molecular-level electron transport, either tunneling or electron hopping. As shown herein, surface hydrophilicity is not the only factor at play. The accessibility channels and tunnels, corresponding to structural vacancies within the protein, are localized at several points and match local high-conductance spots (Figure 2C). This observation provides a new insight to comprehend the complex process of electron transport through proteins.

Importantly, the presence of water molecules from the solvent to these channels does not disturb their conductivity (see image collected in 100 mM KCl in Figure S10c). The magnitude of the tunneling current depends on various factors, including the tip–sample distance, and the presence of bulk water molecules did not hinder the observing of conductance channels. This suggests that tunneling processes can occur through interfacial or bulk water molecules or other species that bridge the electrode and the electrolyte solution, leading to low local barrier heights due to polarization and, possibly, forming localized intermediate states.^41,42^ Solvation can have effects on electron transport in biological systems, depending on the specific system and the nature of the solvation.^43,44^ In the context of redox enzymes, water molecules, despite the insulating property, can act improving the electronic conductance between different parts of the system, affecting ETp as shown in the literature, including metalloproteins, in which the electrostatic interactions is suggested to affect the reorganization energy of ET.^45,46^ Through-water tunneling typically presents a considerably high decay constant (ca. 1.6–1.7 Å^–1^), which might make it counterintuitive how the presence of water did not disrupt observable conductance. Several aspects must be considered here. First is that the through-vacuum decay constant is even higher (3–4 Å^–1^), and electronic transport through shorter distances (comparable to a few peptides) is not too affected by bulk water or protein environments, while still much more efficient than that in a vacuum.^47−49^ Additionally, the high efficiency of hydrogen-bond-mediated ETp has been well documented, with a much greater coupling compared to an analogous carbon–carbon σ-bond system and with a large dependence on the associated interactions and electrostatic forces.^50−53^

The interplay of electrostatics between interfacial water molecules and amino acid portions and ET coupling pathways mediation has been reported for azurin, a blue copper protein.^54^ The presence of internal water in protein pockets was shown to increase tunneling coupling by 1 order of magnitude;^55^ in this sense, they facilitate the transfer of electronic energy and contribute to maintaining currents over longer distances within the system, as also noticed here for conducting channels. Moreover, bulk water in azurin has been shown to not affect the coupling, as only the ordered water molecules closely interacting with the protein that are involved in the minor reorganization energy changes.^48^ The correlation of charge transport and biocatalytic activity has recently been strongly suggested to occur due to the adequate three-dimensional structural arrangements in a NAD^+^–formate dehydrogenase single enzyme study, in which the coupling boosted charge transfer by up to 2100%, leading to high activity and high conductance.^56^ This provides another evidence of the importance of tunnels through the active center containing metallic ions to high conductance.

In conclusion, STM imaging of BOD molecules has provided valuable insights into the electron transfer mechanism in proteins by identifying specific high-conductance channels. While this study demonstrates a correlation between conductance and protein structure, it is important to acknowledge that variations in structural dynamics remain a challenge. To address this, future research should focus on achieving a more precise integration of topographic and current maps, which will be critical for accurately resolving the local conductance in proteins. This work supports recent findings that suggest elevated conductance at contact points in hydrophilic regions. In BOD, electron transport appears to follow multidirectional pathways, with multiple conductance channels that align with accessibility cavities leading to the enzyme’s active center. The local conductance values in BOD reach approximately 15 nS, indicating a highly efficient electron-transport capacity. However, we anticipate that this conductance would be lower for enzymes that are noncovalently bound to surfaces, as the electron coupling will be less effective.

Additionally, we have observed that water does not hinder the formation of conductance channels. On the contrary, water appears to facilitate or maintain tunneling coupling factors, possibly through hydrogen-bond interactions, resulting in enhanced conductance. This observation has significant implications for electrochemical devices that incorporate biomolecules as active components, as understanding the interplay between water, electrolytes, and enzyme surfaces could lead to the design of more efficient bioelectronic systems.

Further investigation is necessary to fully understand the underlying mechanisms governing these multidirectional electron-transfer processes in BOD and to explore their potential applications in bioelectronics, catalysis, and energy conversion technologies.

## Supporting Information Available

Experimental details, cyclic voltametric and current–time curves, FTIR spectrum, optical images, FTIR signal assignment table (PDF)

## Abbreviations

AFM: atomic force microscopy
BA: benzoic acid
BOD: bilirubin oxidase
EDC: 1-ethyl-3-(3-(dimethylamino)propyl)carbodiimide
ET: electron transfer
ETp: electron transport
FFT: fast Fourier transform
HOPG: highly oriented pyrolytic graphite
LDOS: localized density of states
MWCNT: multi-walled carbon nanotubes
NAD: nicotinamide adenine dinucleotide
NHS: *N*-hydroxysuccinimide
ORR: oxygen reduction reaction
STM: scanning tunneling microscopy
TNC: trinuclear cluster.
