# Supplementary material for: Conductance Channels in a Single-Entity Enzyme
Source: J Phys Chem Lett. 2024 Oct 21;15(43):10795–801. doi: 10.1021/acs.jpclett.4c01796 (PMC11533225; doi:10.1021/acs.jpclett.4c01796)
Supplement: Supplementary file 1 — jz4c01796_si_001.pdf [file jz4c01796_si_001.pdf]

Supporting Information
Conductance Channels in a Single-Entity Enzyme

Rafael Neri Prystaj Colombo[a§], Steffane Q. Nascimento[a§], Frank Nelson Crespilho[a*]

[a] 1 São Carlos Institute of Chemistry, University of São Paulo (USP), São Carlos, SP 13566-590, Brazil
*E-mail: frankcrespilho@iqsc.usp.br
§ R.N.P.C. and S.Q.N. contributed equally to this paper

1. Experimental details

1.1 Reagents and materials

Phosphate salts ($NaH_2PO_4.H_2O$, 137.99 g mol^{-1}, >98%; and $Na_2HPO_4.7H_2O$, 168.07 g mol^{-1}, >98%) and isopropanol (C_3H_8O, 60.10 g mol^{-1}, ≥99.5%) were obtained from Synth. NaOH pellets (SKU S5881, 40 g mol^{-1}, >98%), KCl (SKU 746436, 74.55 g mol^{-1}, ≥99%), 1-ethyl-3-(3dimethylaminopropyl) carbodiimidehydrochloride) (EDC, SKU 03450, 191.70 g mol^{-1}, ≥98%), N-hydroxysuccinimide (NHS, SKU 56485, 217.13 g mol^{-1}, ≥98%), sodium nitrite ($NaNO_2$, SKU 237213, 69.00 g mol^{-1}, ≥97%), 2-(N-morpholino)-ethanesulfonic acid (MES, SKU M5287, 195.24 g mol^{-1}, ≥99%), 4-Aminobenzoic acid ($C_{17}H_7NO_2$, SKU A9878, 137.14 g mol^{-1}, ≥97%) were acquired from Sigma-Aldrich. The bilirubin oxidase (EC 1.3.3.5) from *Myrothecium verrucaria* was purchased from Amano Enzyme Inc (BO-3, 2.51 U mg^{-1}, 68 kDa). All aqueous solutions were prepared with deionized water (18 MΩ cm at 25 °C). The HOPG and Pt/Ir tip were acquired from Bruker. All tips were freshly cut as discussed below.

1.2 Apparatus

All STM measurements were performed on a Bruker tunnelling microscope (ECM3/NS6, Bruker Gmbh, Ettlingen, Germany) using a Pt/Ir tip as a probe and ZYB grade HOPG as a substrate and the samples were supported in a Teflon cell sealed with an O-ring and a titanium plate fixed with screws. Vibrational spectroscopy (microFTIR) was performed using a Bruker Vertex 70V spectrometer coupled to a Hyperion 3000 microscope (Bruker Gmbh, Ettlingen, Germany). MicroFTIR spectra were obtained in the reflectance mode with the N_2-cooled Mercury-Cadmium-Telluride detector (MCT). The spectra were recorded from an average of 128 accumulations with a spectral resolution of 4 cm^{-1} and a spectral window of 4000–600 cm^{-1} using an objective lens with 36x magnification.

1.3 Probe preparation

STM measurements were performed using commercial PT-ECM10 platinum-iridium tips (length: 14 mm and diameter 0.25 mm, Bruker Gmbh, Ettlingen, Germany). First, the tips were cleaned in an ultrasonic bath with acetone, isopropyl alcohol, and water for two minutes. The tips were then mechanically sheared at a 45° angle. The tips were screened with an optical microscope, and the best were then selected for usage. For the STM images in liquid, the tips were coated with nitrocellulose resin and dried for 30 minutes at 65°C. This procedure is required to decrease leakage current (which, in the studies carried out, was less than 10 pA) and allowing tunneling currents from the sample only.

1.4 Sample preparation

For HOPG functionalization, as described elsewhere,[1,2] 4-aminobenzoic acid (0.0132 g) was dissolved in 5 mL of deionized water and 5.5 µL concentrated sulfuric acid (Solution A, cooled to 5 °C), then 0.0028 g of sodium nitrite was dissolved in 5 mL of deionized water (Solution B, cooled to 5 °C). After both solutions reached 5 °C, solution B was slowly poured to solution A and stirred in an ice bath for 30 min. 5 mL of the synthesis product was placed in an electrochemistry cell with the HOPG substrate. The potential cycling was performed from 0.6 to -0.6 V vs Ag/AgCl/Cl^{-}_{sat} for 3 scans at a scan rate of 50 mV s^{-1}. The modified HOPG were rinsed thrice with deionized water and dried in a desiccator for 30 min. EDC (0.0345 g) and NHS (0.0209 g) were dissolved in 5 mL of phosphate buffer (0.1 mol L^{-1}, pH 7.2). 10 µL of this mixture was placed on the modified surface device with benzoic acid left to react for 1 hour, then 10 µL BOD solution (1 µg mL^{-1}) diluted in phosphate buffer (0.1 mol L^{-1}, pH 7.2) was added to the electrode and left to 4 °C for 24 hours. The modified HOPG was rinsed with deionized water and dried in a desiccator for 30 min.

1.5 STM measurements

All HOPG-based samples were attached to the electrochemical sample holder with backside electric contact. All Pt-Ir tips were freshly cut prior to use, observed in an optical microscope, and previously tested for quality, being required to achieve atomic resolution for a clean HOPG sample in air. A current offset was applied to make the residual measured tunnelling current typically at a < 5 pA level.

The ECM3 base and NanoScope 6 controller were combined with a 2675AI high-resolution scanner and mounted onto an anti-vibration suspension tripod, **Figure S1**, which comprises a > 20 pound base suspended by three bungee cords stretching over 1 ft the cords' natural length, as this condition is recommended to result in a vertical and horizontal vibration frequencies equal or smaller than 1 Hz; additionally, we coupled a single soft silicone rubber vibration-damping pad under the equipment to further reduce acoustic and subacoustic frequencies propagation from ground, air and through cables. Additionally, a metallic Faraday cage was used to diminish electromagnetic interference, and an acoustic foam hood enclosure is employed to cover the entire equipment (including the Faraday cage) to mitigate environmental noise from air. The system was allowed to stabilize for multiple hours with the tip at ca. 70 nm away from the surface, without scanning; this step is highly valuable to avoid thermal and electronic-related drifts. This setup is noticed to perform drastically better for atomic resolution scale measurements than commonplace setups with anti-vibration air-suspended tables.

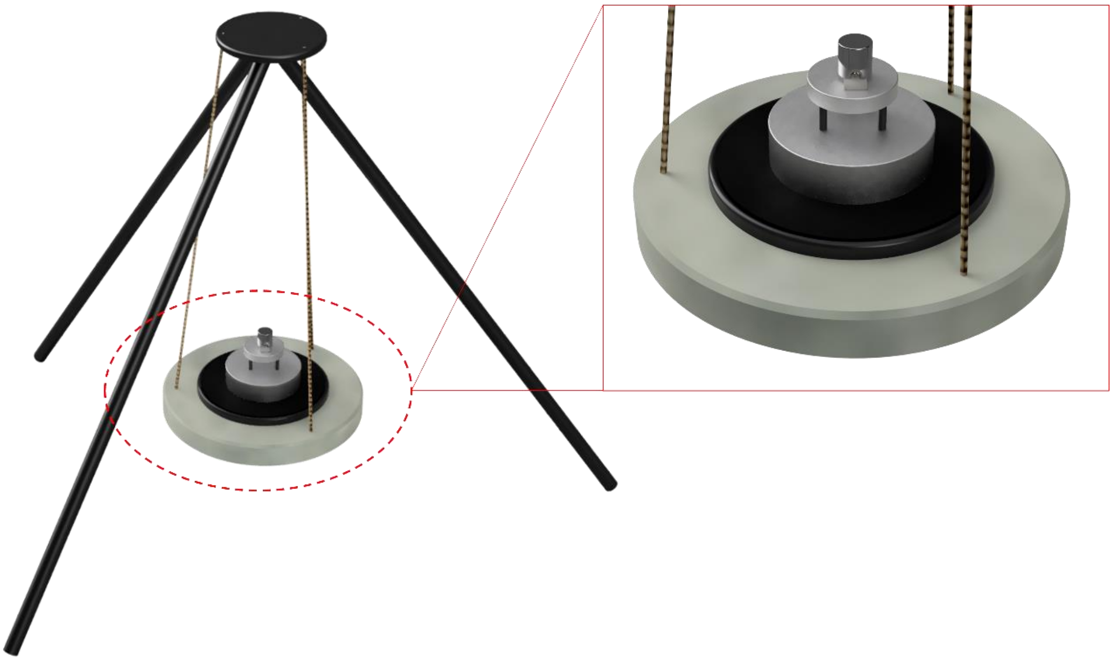

Figure S1. STM tripod schematic. A simplified representation of the suspended STM equipment, by means of a tripod system employing a >20 pound base suspended over 1 ft by bungee cords, leading to < 1Hz horizontal and vertical oscillations.

The STM measurements were performed with indicated bias voltages (V_{bias}) and engaging setpoint (I_s) of ≤ 500 pA, measuring tunnelling currents at a ca. 19.5 μm.s^{-1} rate for HOPG-BA-BOD. No reference electrode is employed as the bias voltage is controlled between the tip and the sample. The resulting micrographs were processed using a first-order line-by-line flattening, in order to correct scale centering and sample tilt (therefore no 2nd order curve fitting), and a low-pass filter through FFT (setpoint r-Period < 5.0 nm), removing spurious high-frequency vertical vectors at the reciprocal space; these wavevectors arise from the scanning motion itself and are easily distinguished from sample features due to the lack of horizontal components (i.e., low amplitude relative to features in the y-axis). Z-axis scale windows were set to aid contrast visualization, not necessarily representing the minimum and maximum currents or conductance from the images.

AFM images were obtained with a FlexAFM from Nanosurf, using Tap190Al-G tips, aluminum coated, 190 kHz nominal resonance frequency, 48 N/m spring constant, ca. 225 μm length, operating at tapping mode, and images were treated with 1st order flattening and 2D-FFT to remove background noise.

1.6 Protein Modeling

The BOD protein data was obtained from PBD 2XLL, and set to display the external surface with two color schemes, (i) with α-helix regions in cyan, β-sheet regions in red, random structures in magenta, and lysine or arginine rich sites in yellow; (ii) with most hydrophilic sites in white and most hydrophobic sites in red.[3] The accessibility cavities to/from the TNC and T1 pockets were geometrically determined through Voronoi Diagrams-based algorithm using Caver 3.0,[4] and plotted as a meshed space; mesh colors randomly set to aid visualization. Purely internal or improbable cavities are not shown.

2. Micro-FTIR Analysis

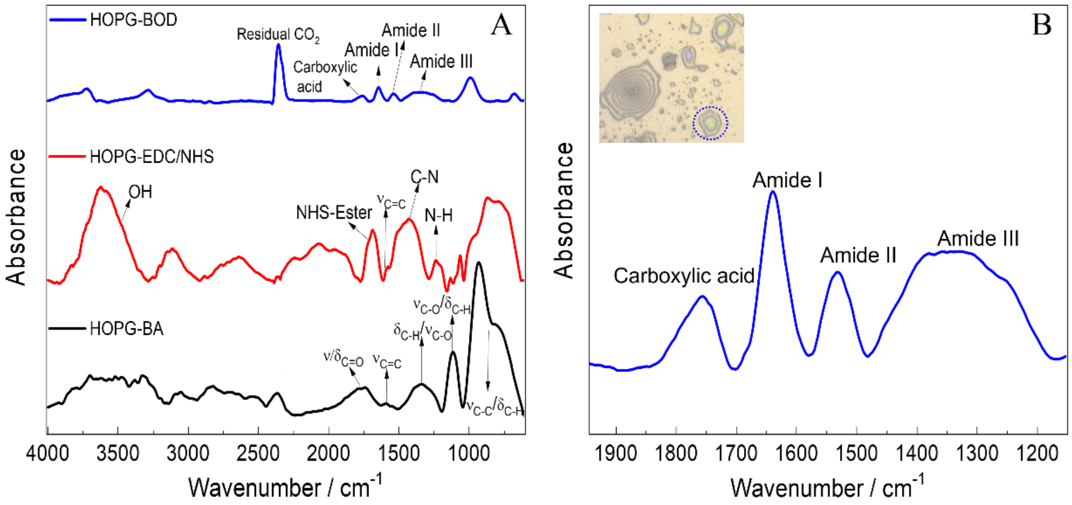

Figure S2. μFTIR spectra. A) FTIR spectra of HOPG-BA (black), HOPG-EDC/NHS (red) and HOPG-BOD (blue). B) Zoomed-in spectrum of HOPG-BOD in the Amides region. *Inset:* optic micrograph (36X objective) of HOPG-BOD.

3. HOPG Analysis

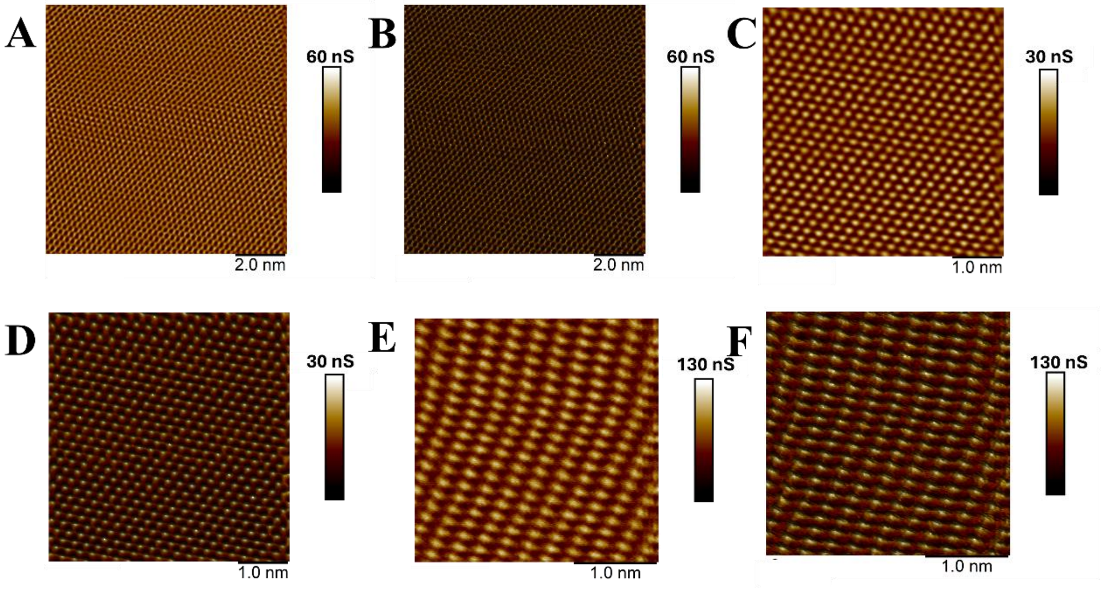

Figure S3. STM images of bare HOPG. Constant height micrographs and apparent conductance of A) HOPG 10 nm scan window, V_{bias} = 20 mV, I_s = 1.3 nA. B) HOPG 10nm scan window (post-FFT). C) HOPG 5 nm scan window, V_{bias} = 20 mV, I_s = 2.2 nA. D) HOPG 5 nm scan window (post-FFT). E) HOPG 5 nm scan window in 0.1 mol.L^{-1} KCl electrolyte, V_{bias} = 10 mV, I_s = 1.3 nA. F) HOPG 5 nm scan window in electrolyte (post-FFT).

4. **HOPG-BA and HOPG-BA-EDC/NHS Analysis**

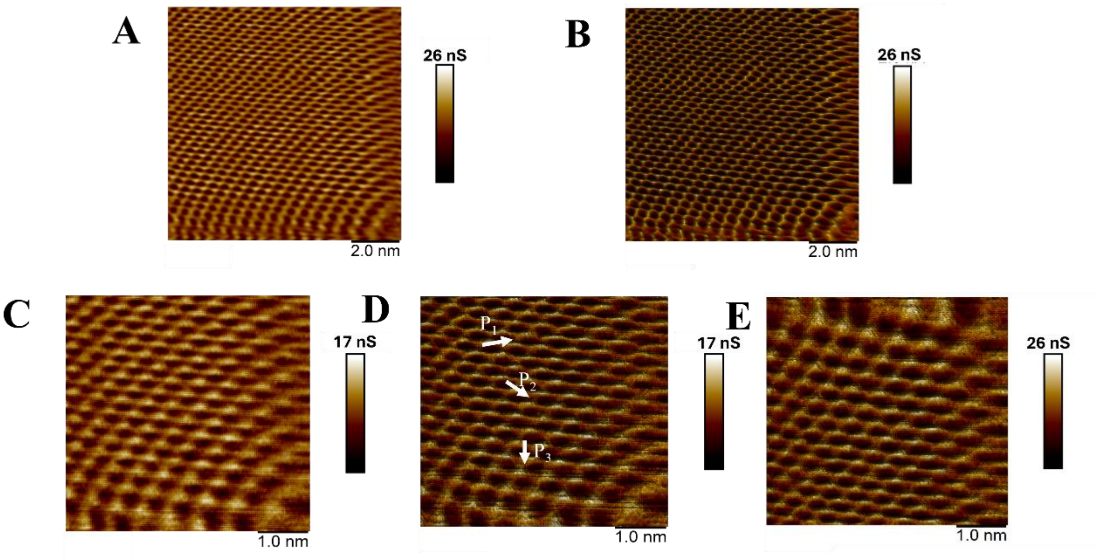

Figure S4. HOPG Modification with benzoic acid. Constant height STM micrographs of A) HOPG-BA 10 nm scan window, V_{bias} = 100 mV, I_s = 9 nA. B) HOPG-BA 10 nm scan window (post-FFT). C) HOPG-BA 5 nm scan window, V_{bias} = 150 mV, I_s = 8 nA. D) HOPG-BA 5 nm scan window (post-FFT). E) HOPG-BA 5 nm scan window in 0.1 mol.L^{-1} KCl electrolyte (post-FFT), V_{bias} = 100 mV, I_s = 2.6 nA.

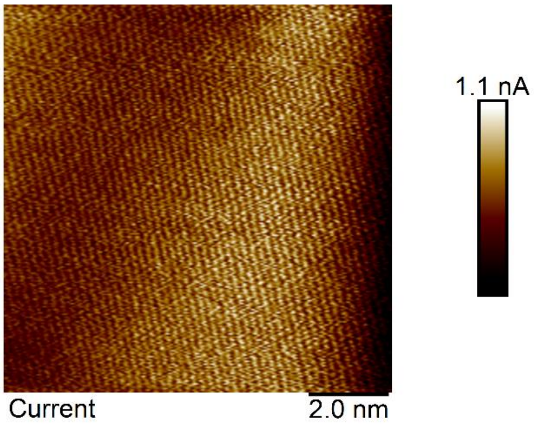

Figure S5. HOPG-BA-EDC/NHS modification. Typical STM current image of a HOPG sample modified with the benzoic acid layer through electrochemistry, followed by EDC-NHS, without proteins. V_{bias} = 50 mV, I_s = 1.1 nA, without KCl electrolyte.

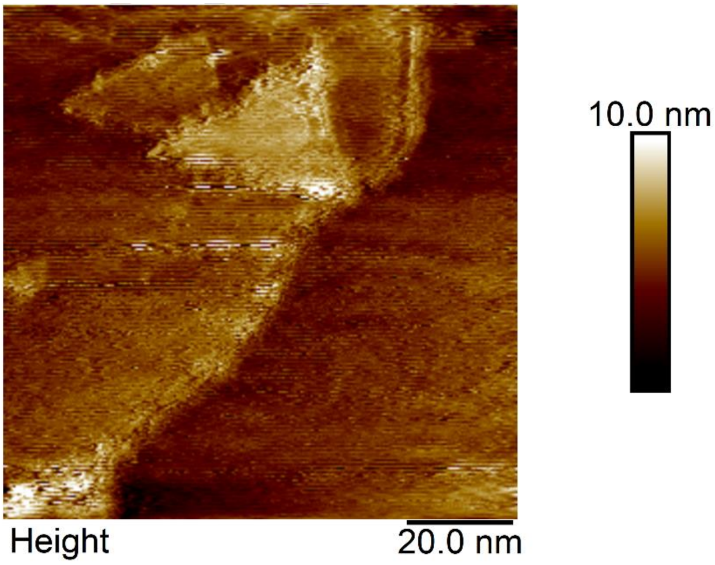

Figure S6. BOD adsorption attempt onto HOPG. STM micrograph (height profile) of HOPG after BOD adsorption, followed by rinsing to remove weakly interacting proteins. No visible or distinguishable proteins are seen in contrast to the samples with covalently linked proteins. V_{bias} = 200 mV, I_s = 4.4 nA.

5. Accessibility Tunnels to BOD’s Active Centre

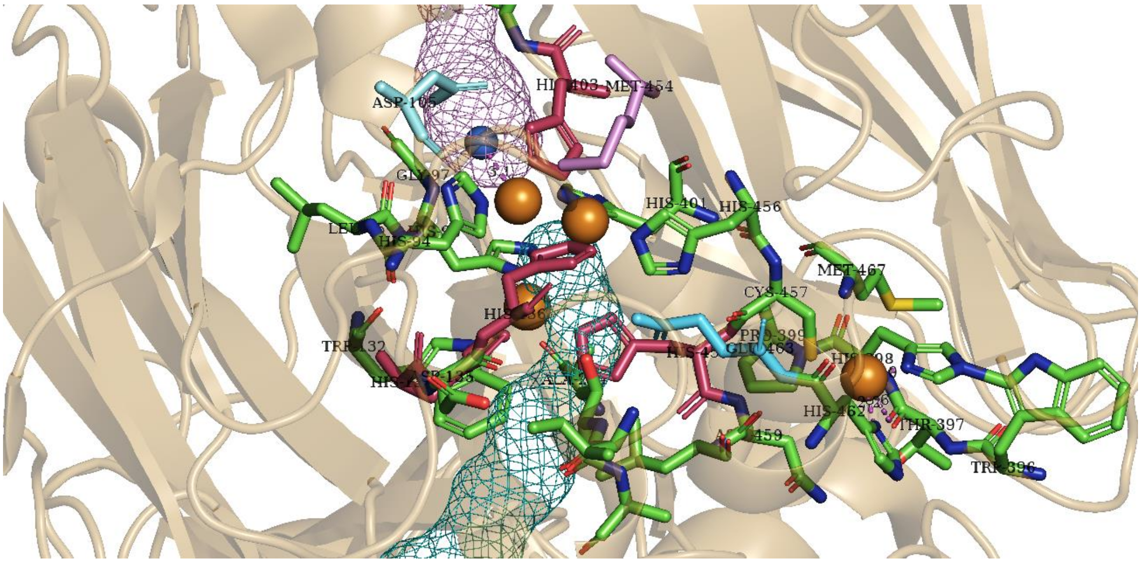

Figure S7. Accessibility tunnels to BOD’s TNC. TNC pocket with the three copper ions (orange spheres), the main structural amino acid residues, and the accessibility tunnels in purple and cyan; a water is seen within the purple tunnel (blue sphere), which is the main entrance for water oxidation reaction. Image obtained from ref. [5] with permission from Wiley.

6. HOPG-BOD Analysis

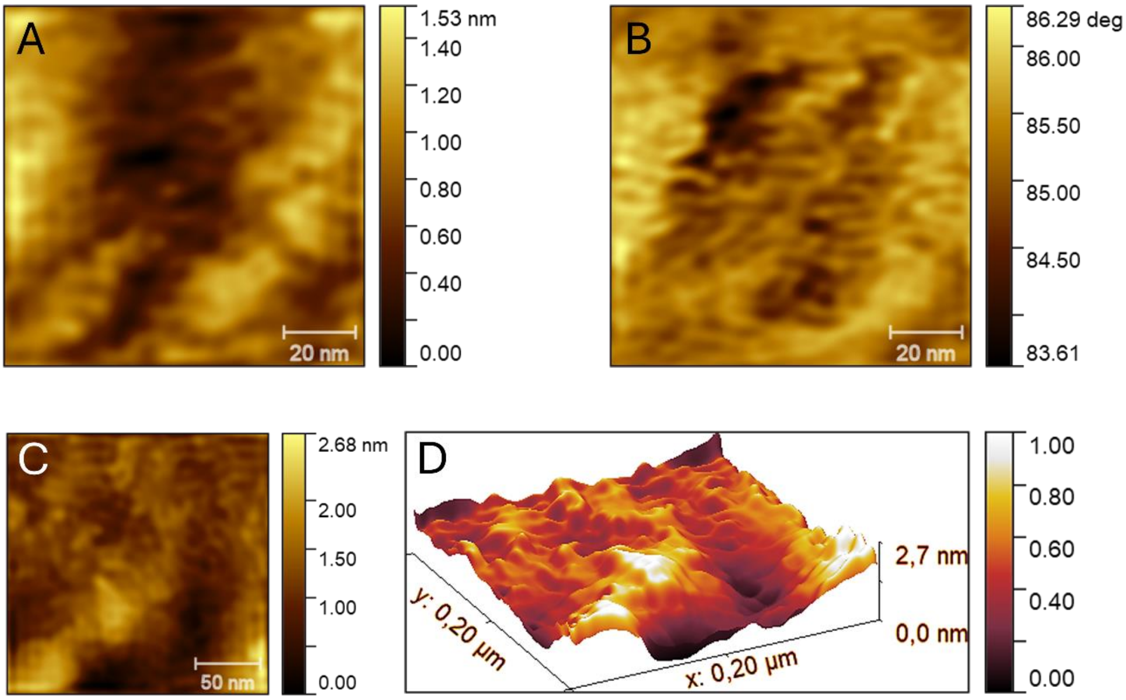

Figure S8. AFM scans of the HOPG modified with BA and BOD. A) 100 nm scan window topography and B) phase images. C) A 200 nm scan window topography micrograph. D) 3D representation of the surface in a 200 nm x 200 nm range. Despite showing present features, tip resolution is not good enough for trustful numerical heigh information or for depicting precise contours of the features.

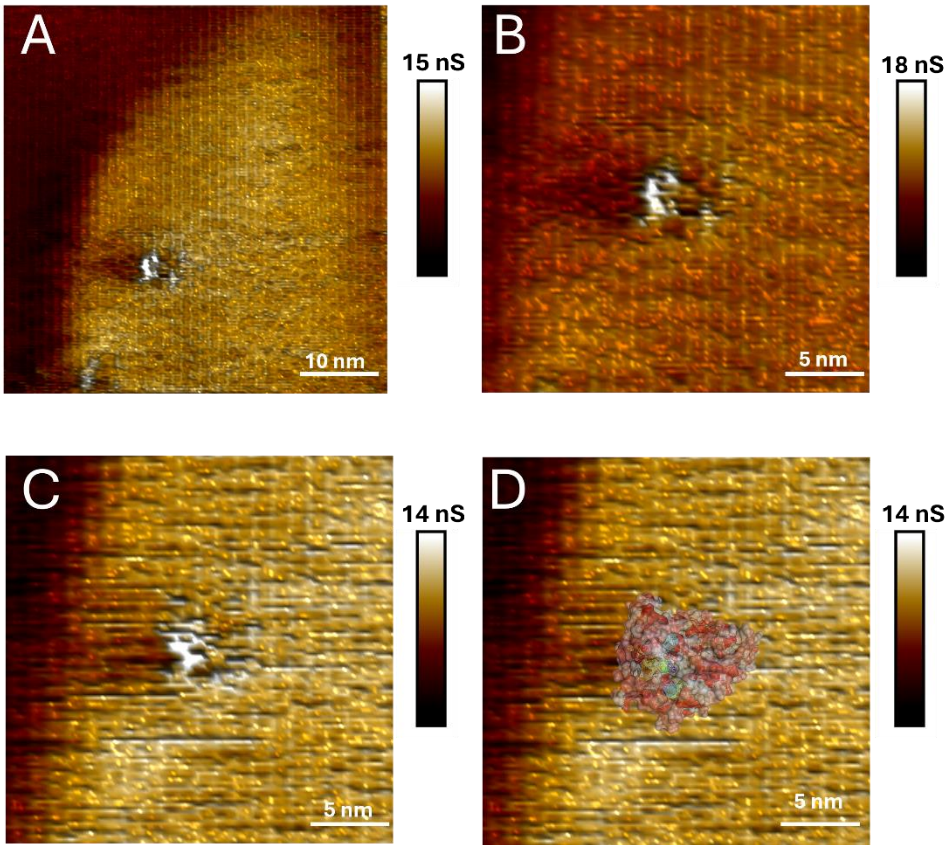

Figure S9. Additional examples. A) One isolated BOD protein in a wider 50 nm scan window, I_s = 3.9 nA, V_{bias} = 100 mV. B) Zooming in, the protein shows two regions with higher conductance, due to the cavities leading to the Cu-containing active centre and regions with higher hydrophobicity; a distortion can be seen, probably due to thermal drift or environmental noise, I_s = 3.9 nA, V_{bias} = 100 mV. C) Another example, reaching conductance close to 15 nS matching external hydrophilicity regions and a high-conductance spot over the accessibility channel to the multicopper centre, I_s = 3.9 nA, V_{bias} = 100 mV. D) Overlayed structure with emphasis on the active centre accessibility cavities (meshed region). Images collected without electrolyte.

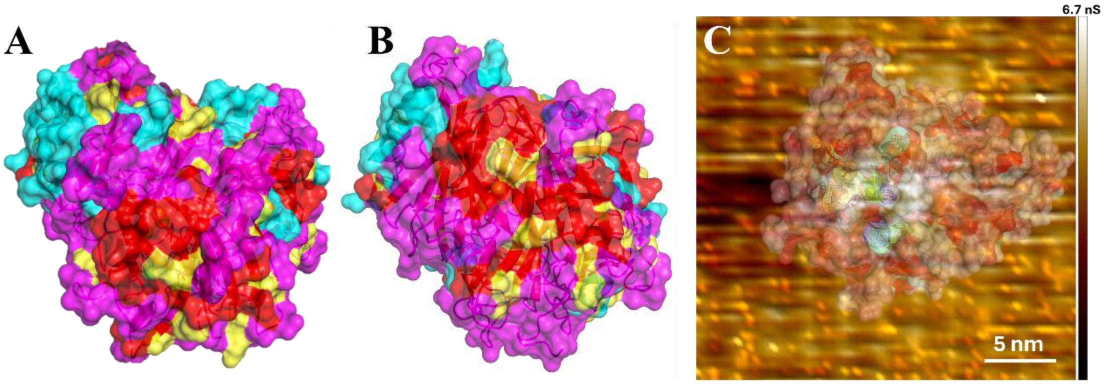

Figure S10. HOPG-BOD. A) Side view from the orientation in which the majority of coupling-sensitive residues are located at the bottom, represented in yellow. B) Inferior view, emphasizing the concentration of the viable crosslink residues in yellow; this region is putatively in preferential contact to the surface due to the EDC-NHS linking process. C) STM micrograph of an isolated BOD protein with structural overlay, with

main channels connecting the external site to the TNC and Cu_{T1} regions exhibiting higher conductance. Micrograph acquired in the presence of 0.1 mol.$^{L-1}$ KCl electrolyte; I_s = 8.5 nA, V_{bias} = 100 mV.

[1] L. J. A. A. Macedo, F. C. D. A. D. A. Lima, R. G. Amorim, R. O. Freitas, A. Yadav, R. M. Iost, K. Balasubramanian, F. N. Crespilho, *Nanoscale* **2018**, *10*, 15048.

[2] Y.-C. Liu, R. L. McCreery, *J. Am. Chem. Soc.* **1995**, *117*, 11254.

[3] D. Eisenberg, E. Schwarz, M. Komaromy, R. Wall, *J. Mol. Biol.* **1984**, *179*, 125.

[4] E. Chovancova, A. Pavelka, P. Benes, O. Strnad, J. Brezovsky, B. Kozlikova, A. Gora, V. Sustr, M. Klvana, P. Medek, L. Biedermannova, J. Sochor, J. Damborsky, *PLoS Comput. Biol.* **2012**, *8*, e1002708.

[5] G. C. Sedenho, I. T. Neckel, R. N. P. Colombo, J. C. Pacheco, T. Bertaglia, F. N. Crespilho, *Adv. Energy Mater.* **2022**, 2202485.
